# Focused Liquid Pinching in Coaxial Drop Capsule Generation

**DOI:** 10.1021/acs.langmuir.5c06135

**Published:** 2026-02-18

**Authors:** Nilofar Taraki, A. Said Ismail

**Affiliations:** School of Engineering and Materials Science, 4617 Queen Mary University of London, London E1 4NS, U.K.

## Abstract

The pinching dynamics of an inviscid inner drop in a coaxial pendant drop structure have been investigated here both experimentally and numerically. The thinning rate of the inner drop, when it pinches in an inertial regime in this configuration, is found to be faster than that of a single drop pinching in a liquid medium. This is attributed to a focusing of the flow between the inner and outer drop interfaces induced by the contraction of the outer drop during its own pinching process. Our results reveal that this focusing effect increases dramatically when the ratio of the inner to outer nozzle radii, *R̃*, in a coaxial nozzle configuration exceeds 0.67. Beyond this value, the thinning rate becomes dependent on the outer nozzle size. Furthermore, the difference between the minimum neck radii of the inner and outer drop, denoted as Δ*h*_min_, serves as a reliable predictor of the inner drop’s thinning rate, even for different outer liquid viscosities, and helps identify the conditions under which the satellite droplets form. Satellite droplets are observed when the outer drop squeezes the inner filament above its minimum neck location, which occurs when Δ*h*_min_ ranges between 0.3% and 1% of the capillary length.

## Graphical abstract

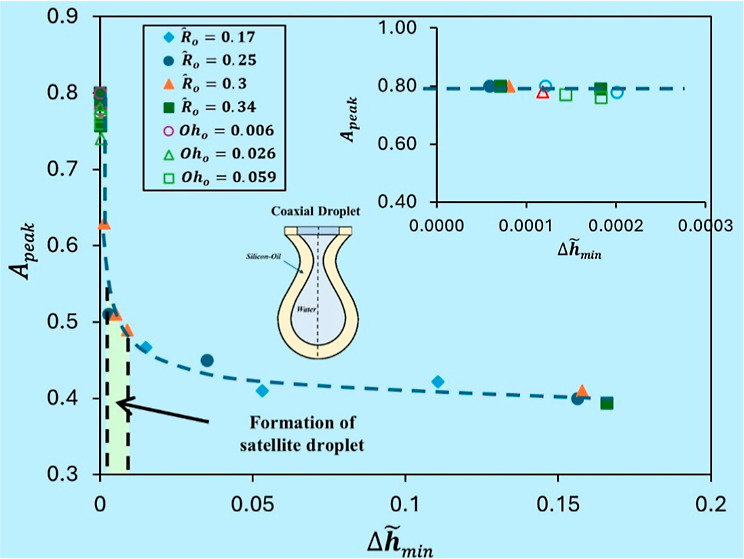

## Introduction

A capsule, in which a drop is trapped inside another immiscible liquid drop, appears vital in many industries, such as biological applications,^1–4^ drug administration,^5,6^ pharmaceuticals,^7,8^ food,^9^ and medical diagnosis.^10^ Scientists have been working on optimizing coaxial flow devices^11,12^ to create more controlled capsules that allow for more precise usage in the aforementioned applications. A common process to create the capsule in these devices is the pinching of the coaxial flow structure into coaxial drops. Drop pinch-off has been carefully studied over the last decades, covering aspects such as the thinning of the formed liquid filament neck prior to its pinching,^13^ the length of the liquid filament,^14^ the pinching angle,^15^ the formation of satellite droplets during the pinching process^16^ and the more global geometrical effect, such as the nozzle size and angle.^17^

A set of mathematical models have been developed to predict the pinching dynamics of a single drop in different regimes,^18–20^ namely the inertial, viscous, and inertial-viscous regimes. Within the inertial regime, the inertia is balanced with the capillary force, and the viscous force is considered to be negligible. The minimum necking radius, *h*_min_, in the inertial regime, decreases with time following the scaling ${\mathrm{h̅}}_{\min}=A\tau^{2/3}$ , where ${\mathrm{h̅}}_{\min}$ is the dimensionless minimum necking radius and *A* is the inertial prefactor, which reflects the thinning rate of the neck and exhibits a nonmonotonic variation over time. τ is the dimensionless time defined by the equation τ = (*t*_*b*_ – *t*)/*t*_*c*_, where *t*_*b*_ is the breakup time, *t* is the time step, and $t_{c}=\sqrt{\left(\rho R_{n}^{3}/\sigma \right)}$ is the capillary time. Here ρ is the fluid density, *R*_*n*_ is the nozzle radius, and σ is the surface tension. The maximum value of *A*, or the peak, is thought to be around 0.7 for a single drop pinching in air;^21^ however, Deblais et al.^22^ found that *A* is not universal and can be significantly altered by changing the nozzle size from which the drop is suspended or by making small changes to the viscosity. For drop pinching in another liquid, another study found that *A*_peak_ can vary between 0.4 and 0.7, based on the external liquid viscosity.^23^

The formation of satellite droplets after the pinching of the liquid drop filament has been drawing the attention of researchers. This is primarily because many industrial applications, such as inkjet printing and microfluidic flow systems, deem the presence of the droplets as being unwanted and compromising the device’s precision. Recent research, conducted by Li et al.^24^, provided theoretical proposals for suppressing the production of said satellite droplets by manipulating the influence that the slenderness of the liquid filament has upon the final destination of the satellite droplet. It was concluded that if the filament slenderness exceeded 1.81, the satellite droplet would be recollected or absorbed back into the upper mound of the fluid.

All of the work discussed thus far focused only on the pinching dynamics of a single drop. In this article, the pinching dynamics of coaxial liquid drop structures are investigated experimentally and numerically by dripping coaxial pendant drops from a coaxial nozzle. We focus our work mainly on understanding how the inner drop pinches off when the thinning is happening in the inertial regime and aim to reveal the conditions in which satellite droplets are produced and potentially inhibited in such a structure.

## Experiments and Numerical Simulations

The experimental setup shown in Figure 1a is built to generate, and eventually pinch, coaxial pendant drops emerging from a coaxial nozzle. As illustrated in Figure 1b, the coaxial device includes a junction connected to two plastic tubes: a plastic tube from the side, which carries the outer fluid, and a plastic tube on the top, which carries the inner fluid straight through. These tubes are in turn connected to two 10 mL syringes mounted on syringe pumps to independently control the flow rates of the inner and the outer fluids, denoted as *Q*_*i*_ and *Q*_*o*_, respectively. Both fluids are injected at very low flow rates, less than 2 mL/min, to ensure that the drop pinches off under the effect of its weight only. In this study, two immiscible liquids, water, and oil, are injected through the outer and inner nozzles and then allowed to pinch-off in the air, as shown in Figure 1c. The pendant drop method is used to measure the surface tension of the oil and the interfacial tension of the water and oil. A high-speed camera (Photron Fastcam Mini AX200) is used to capture the pinch off process of the inner and outer filaments. Consistent lighting is maintained using an optical diffuser to ensure an accurate detection of the drop interface in all images.

**Figure 1:**
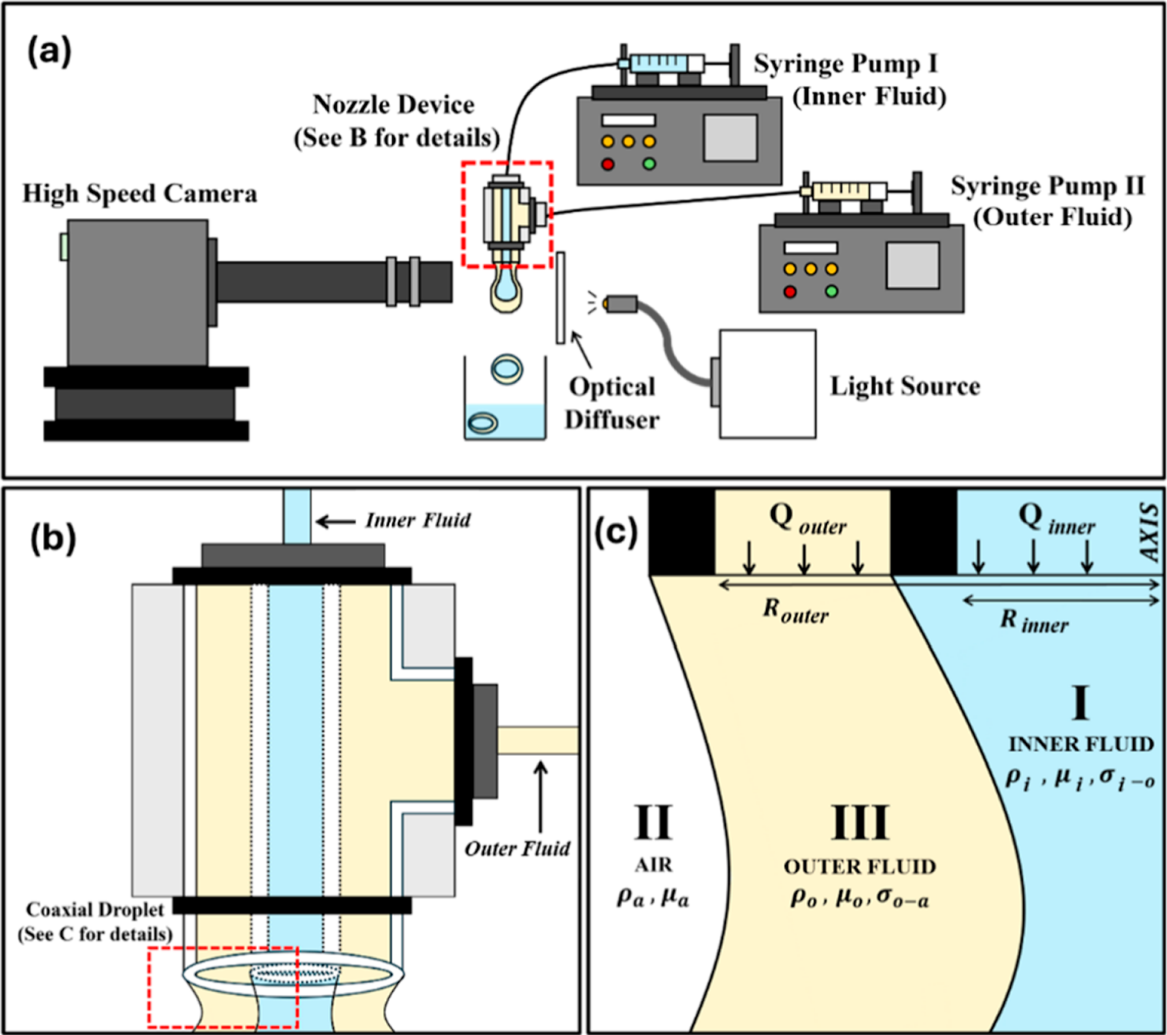
Diagrams showing (a) experimental setup, (b) zoom-in into the configuration of the coaxial nozzle, and (c) further zoom-in into the coaxial nozzle device’s exit showing the order of the fluids in this three-phase configuration.

A series of experiments are conducted using the coaxial nozzle with an inner nozzle radius, *R*_*i*_, of 0.45 mm and an outer nozzle radius, *R*_*o*_, of 1.5 mm. Figure 2 shows experimental images of the coaxial pinching process when oil is injected through the inner nozzle and water through the outer nozzle, with an inner-to-outer flow rate ratio, *Q* = 0.5, where *Q* = *Q*_*i*_/*Q*_*o*_. As the inner and outer drops expand, the inner drop eventually forms a neck, as shown in the images in Figure 2 by the slender filaments that join the top and bottom of the drop. This neck thins progressively until it is pinched off, which is then followed by the thinning and eventual pinching of the outer drop. The images in Figure 2 highlight certain optical limitations in the experiment. The external drop acts as a convex lens, which focuses the light at the center and makes it difficult to accurately detect the inner drop interface, especially when the inner and outer interfaces become closer at higher inner-to-outer nozzle ratio *R*.

**Figure 2:**
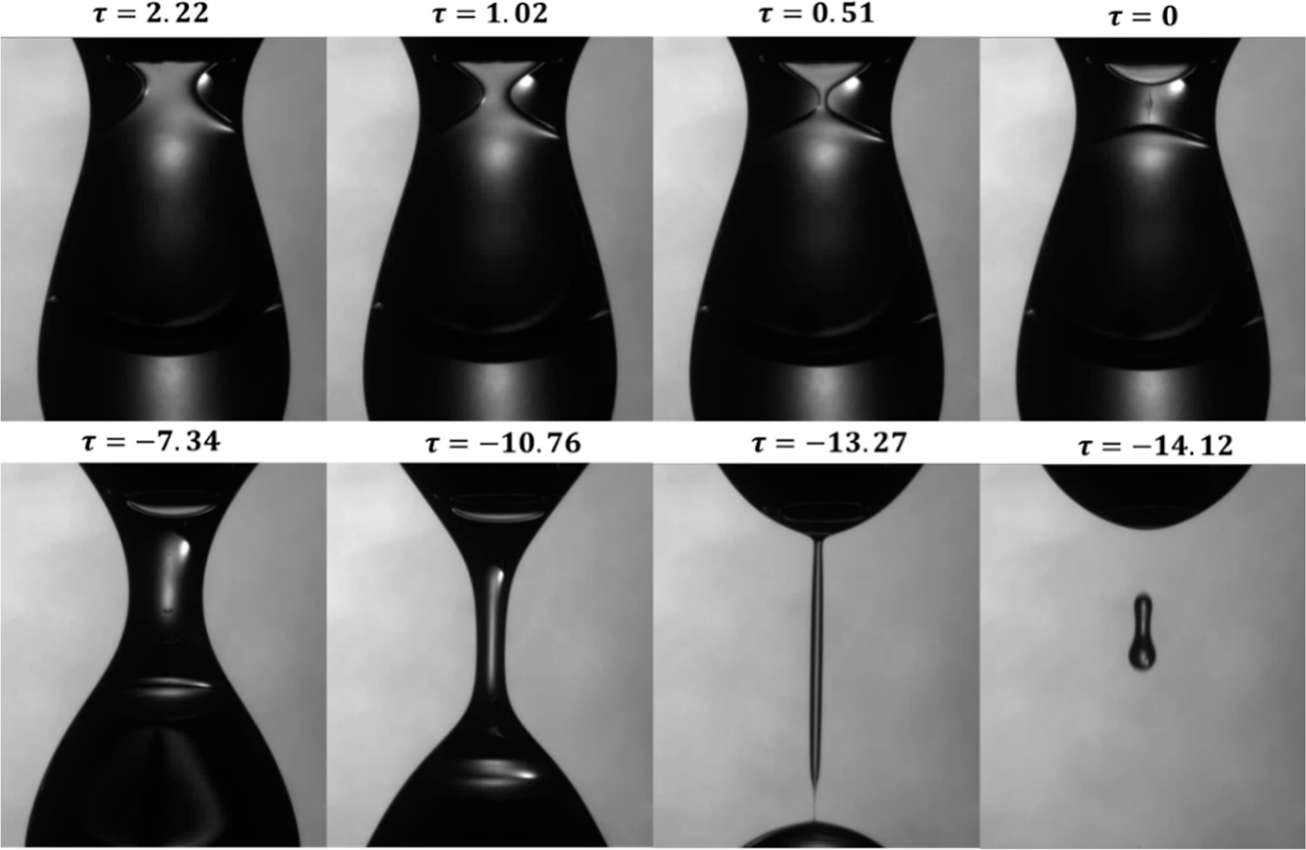
Experimental images showing the coaxial pinching sequence of sunflower oil (inner fluid) surrounded by water (outer fluid), pinching-off in air, where the inner nozzle radius, *R*_*i*_ = 0.45 mm and the outer nozzle radius, *R*_*o*_ = 1.5 mm.

To tackle this limitation, a multiphase model is developed using open-source software Gerris. The model simulates the coaxial pinching process to accurately track the inner interface during the thinning, even at a high value of *R*. An axisymmetric computational domain is considered, and the fluids used are assumed to be incompressible and immiscible; thus, the simulation relies on solving the Navier–Stokes equation with surface tension and the continuity equation shown below, respectively.

3.1 $$\rho \left(\partial \text{\_}\left(\right)tv+v\cdot \nabla v\right)=-\nabla p+\nabla \cdot \left(2\mu D\right)+\sigma \kappa \delta_{s}n$$

3.2 ∇·v=0

where ***v*** and *p* represent the velocity field and pressure distribution, respectively, while ρ and μ represent the fluid density and the dynamic viscosity, respectively. The deformation tensor, ***D***, is dictated by the equation ***D*** = (∇ ***v*** + ∇ ***v*** ^*T*^)/2. The nature of the surface tension coefficient, σ, functioning along the interface is represented by the Dirac distribution function, δ_*s*_. κ and ***n*** denote the radius of curvature and perpendicular unit vector of the interface, respectively. To track the interface position of the inner and outer drop, volume fractions *c*_*i*_ (***x***, *t*) are calculated using the advection equation below.

3.3 $$\frac{\partial c_{i}}{\partial t}+v\cdot \nabla c_{i}=0$$

The subscript *i* = 1,2 with the numbers denotes the inner-to-outer drop fluid fraction and air-to-outer fluid fraction, respectively. This equation is solved using a piecewise-linear geometrical Volume-Of-Fluid (VOF) scheme.^25^ The volume fractions *c*_1_ and *c*_2_, which vary between 0 and 1, are then used to estimate the values of the local viscosity and density in the three-phase computational domain by using the following equations

3.4 $$\mu \left(c_{1},c_{2}\right)=c_{1}\mu_{i}+c_{2}\mu_{a}+\left(1-c_{1}-c_{2}\right)\mu_{o}$$

3.5 $$\rho \left(c_{1},c_{2}\right)=c_{1}\rho_{i}+c_{2}\rho_{a}+\left(1-c_{1}-c_{2}\right)\rho_{o}$$

where μ_*i*_, μ_*o*_, and μ_*a*_ represent the dynamic viscosity of the inner fluid, outer fluid, and the surrounding air, respectively, while ρ_*i*_, ρ_*o*_, and ρ_*a*_ represent the density of the inner fluid, outer fluid, and the surrounding air, respectively. The combination of variables in eqs [Disp-formula eq3_4] and ([Disp-formula eq3_5]) allows for the correct fluid properties to run in their designated regions.

Figure 3 shows the computational domain that is used, with dimensions of 20 × 10 mm. The boundary conditions on the left side of the domain were divided into three parts, as shown in Figure 3: the inlet velocity for the first fluid and the inlet velocity for the second fluid, which together mimic a coaxial nozzle outlet flow. The remainder of the left side of the domain, along with the top side, consisted of a solid wall with no-slip boundary conditions, where the velocity components were set to 0. On the right-hand side, the outflow boundary condition is set, in which the pressure and the velocity gradient are set to zero.

**Figure 3:**
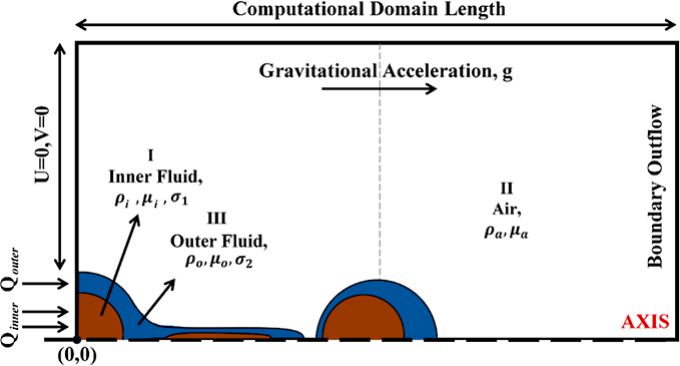
Schematic of the three-phase axisymmetric computational domain that was used to simulate the coaxial drop pinching.

An adaptive mesh gradient is used to save computational costs and time while simulating at a high enough refinement to precisely capture the delicate necking filament. The reader is referred to Popinet’s paper^26^ about the used adaptive mesh technique for more details. A mesh convergence study is conducted to identify the appropriate refinement level that ensures consistency of the fluid interface across meshes. Since our main focus within this work is to study the thinning rate during the pinching while in the inertial regime, we examine the inertial prefactor, *A*, against varying levels of refinement. *A* was determined using the approach described by Li & Sprittles,^27^ by extracting the slope from the relationship (*h*_min_/*R*_*i*_)^3/2^ = *A* ^3/2^τ. This technique avoids the need to calculate the precise moment when *h*_min_ = 0. From Figure 4 it becomes clear that the temporal inertial prefactor converges above level 11 and the characteristic peak inertial prefactor, *A*_peak_, (indicated by the black cross in Figure 4), becomes almost independent of the mesh size. Based on this analysis, we use level 12 mesh refinement throughout the breadth of our study to ensure accurate results while maintaining efficient computational costs. The mesh size Δ = 0.01/2^Level^. For level 12, this gives a mesh size of approximately 2.4 μm near the liquid interface.

**Figure 4:**
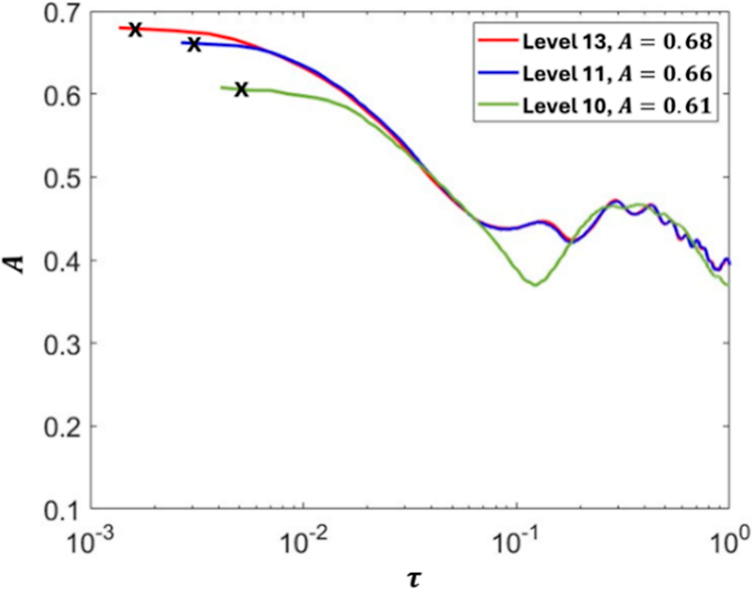
Comparison of the temporal inertial prefactor for different mesh refinement levels; the black markings shown by “*x*” represent the peak values of the inertial prefactor, *A*_peak_.

In order to validate the computational model, a side-by-side comparison is performed between the experimental images of the coaxial drops and their corresponding computational simulations. The inner fluid used is silicone oil (100cSt), with a density of 960 kg/m^3^, and the outer fluid used is water. The values of the inner and outer radii were 0.45 mm and 1.5 mm, respectively, with *Q* = 1. The interfacial tension between the silicone oil­(100cSt) and water was measured experimentally to be 0.037 N/m using the pendant drop technique. The experimental and computational images show excellent agreement, as seen in Figure 5, which demonstrates that the model accurately predicts the temporal evolution and thinning behavior of coaxial drops.

**Figure 5:**
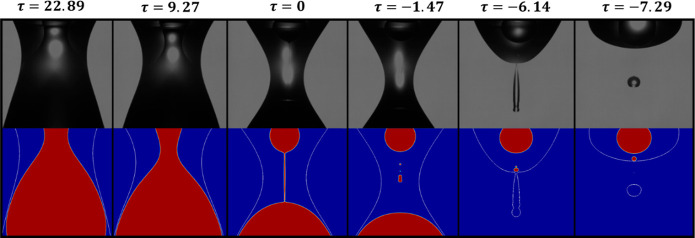
Computational images (bottom) and their experimental counterparts (top) of the pinching process. The breakup time used to calculate τ is the one for the inner drop.

## Results and Discussion

Within the confines of this study, we primarily focused on the pinching of the inner drop in the coaxial structure. We fix both the inner and the outer flow rate to *Q*_*i*_ = 1 mL/min and *Q*_*o*_ = 0.2 mL/min, and we use water as the inner fluid and silicone oil­(10cSt) as the outer fluid. The density of the silicone-oil­(10cSt) used is 930 kg/m^3^, and the interfacial tension between the water and oil is 0.023 N/m, whereas the surface tension between the oil and air is 0.0185 N/m. To understand the dynamics of this pinching, we first run numerical simulations to compare the pinching of the water inner drop in a coaxial nozzle configuration with the pinching of a single water drop dripping from a single nozzle in a liquid pool of silicone oil (10 cSt) (see configurations in Figure 6, sub-picture). The values of the inner radius of the coaxial nozzle and the radius of the single nozzle are kept the same to ensure comparable results. In Figure 6, the temporal distribution of the inertial prefactor, for nozzles with three different sizes, shows that the value of *A* for the inner drop in the coaxial configuration is higher in all three cases than that of the single ones, reflecting a faster thinning rate.

**Figure 6:**
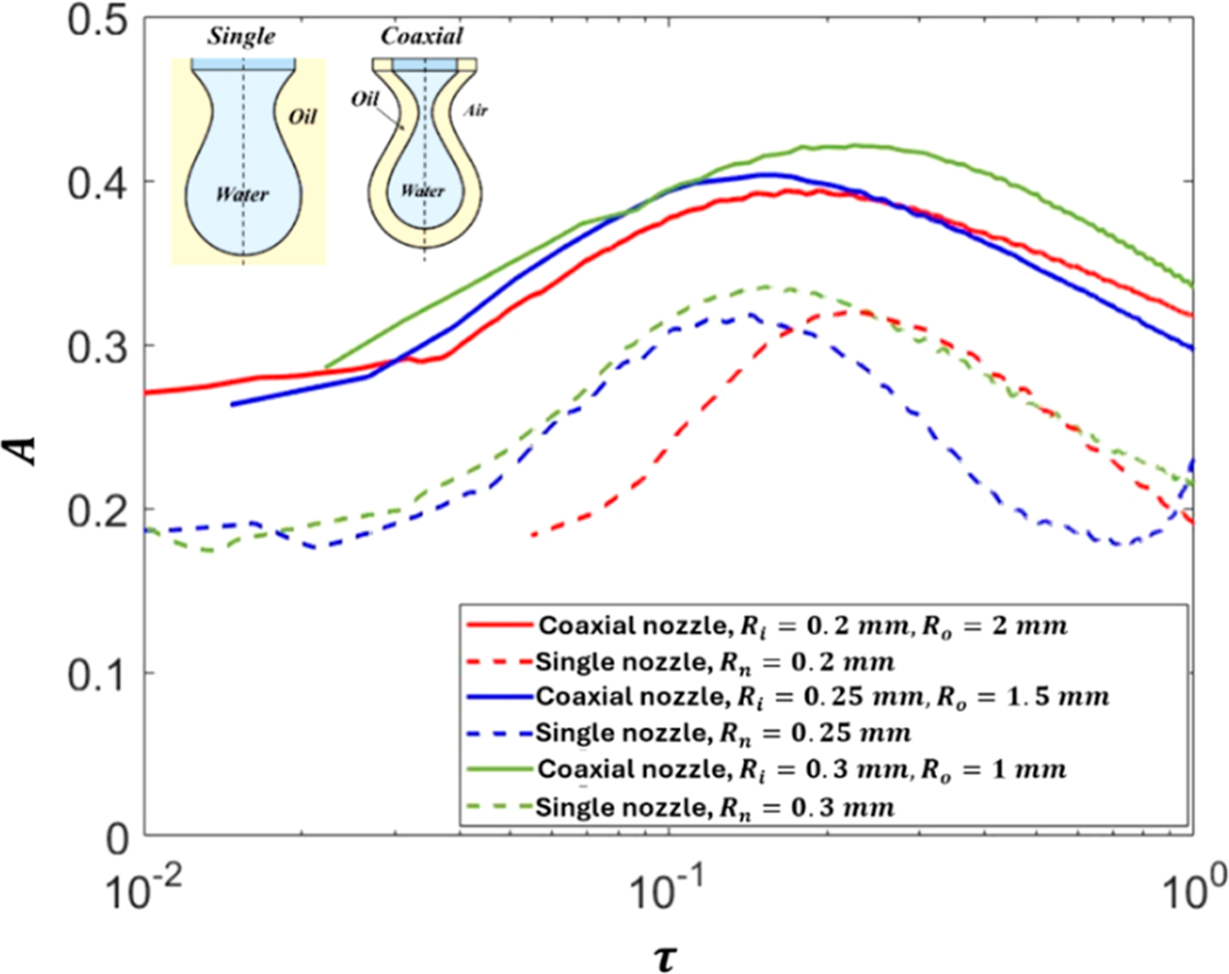
Thinning of the drop filament for single and coaxial droplet cases represented as A vs τ.

To have a more quantitative comparison, we represent the peak inertial prefactor, *A*_peak_, for each configuration against their nozzle sizes. As shown in Figure 7a, for a single nozzle, *A*_peak_, and hence the thinning rate increases with the nozzle size as long as the nozzle radius <1 mm. Above 1 mm, the pinching mechanism behaves as a local phenomenon and becomes independent of the nozzle size. Moreover, the thinning rate, represented in the form of *A*_peak_, is higher in the coaxial configuration for all cases. Additionally, as *R*_*i*_ increases in the coaxial configuration, the thinning rate increases slowly for *R*_*i*_ < 1 mm and increases dramatically for *R*_*i*_ > 1 mm, as it approaches *R*_*o*_ in size. If one were to look at the coaxial configuration, shown by Figure 7b, we can see that the interface of the external drop is far away from the inner drop neck, and thus, it is expected that the thinning rate should not be influenced by the external interface. However, as seen in Figure 7b, there are points of contact between the two fluids along their interfaces. As the external drop grows bigger in size, its weight increases, which pulls the inner drop down, leading to a faster thinning rate in comparison to the single drop pinching. This observation can be verified by comparing the inner drop in the coaxial nozzle configuration with a single drop pinching in a single-nozzle configuration. Figure 7c demonstrates that the inner-drop thread in the former case is more elongated than that in the latter, which is attributed to the increased pull of gravitational force.

**Figure 7:**
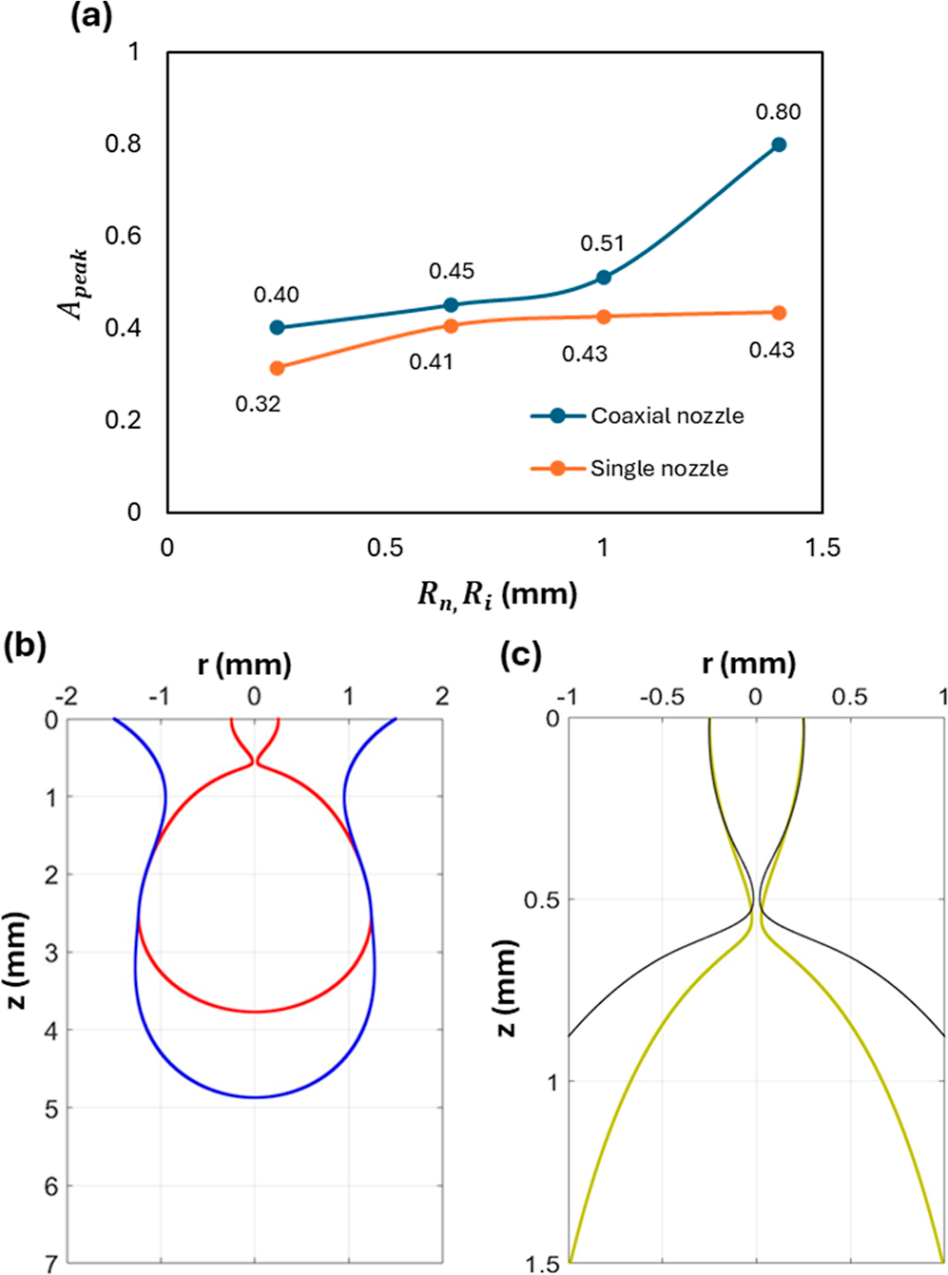
(a) Peak inertial prefactor as a function of the nozzle size for both single and coaxial cases (*R*_0_ for all the coaxial cases is the same at 1.5 mm), (b) coaxial drop pinching at the moment of *A*_peak_ when *R*_*i*_ = 0.25 mm and *R*_*o*_ = 1.5 mm, and (c) inner drop in the coaxial nozzle (thick green line) compared with a drop in single nozzle (thin black line).

To understand this nonlinear thinning behavior in the coaxial configuration, we run simulations of the coaxial pinching with different inner and outer nozzle radii. Figure 8 shows the inner drop *A*_peak_ versus the dimensionless inner nozzle radius ${\mathrm{R̃}}_{i}=R_{i}/L_{c}$ for different dimensionless outer nozzle radii ${\mathrm{R̃}}_{o}=R_{o}/L_{c}$ , where the capillary length $L_{c}=\sqrt{\sigma_{io}/\left(\rho_{i}-\rho_{o}\right)g}$ and σ_*io*_ is the interfacial tension between the inner and outer liquids. The general trend is that *A*_peak_ increases slowly with the inner nozzle for ${\mathrm{R̃}}_{i}<$ 0.17. Above 0.17, *A*_peak_ increases rapidly with a rate dependent on the outer nozzle before it saturates at *A*_peak_ = 0.8.

**Figure 8:**
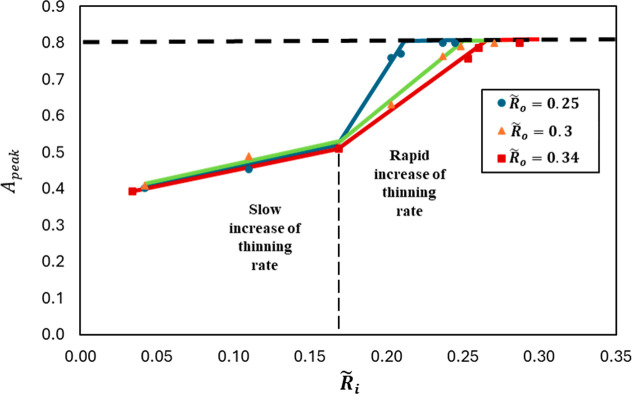
Peak inertial prefactor for the inner drop as a function of the inner nozzle size for different outer nozzle sizes.

For ${\mathrm{R̃}}_{i}<$ 0.17, *A*_peak_ increases slowly with ${\mathrm{R̃}}_{i}$ independently of ${\mathrm{R̃}}_{o}$ . This increase in *A*_peak_ is attributed to a focusing effect introduced when the external interface approaches the internal one, as it can be seen in Figure 9. This focusing effect is an increase in the flow between the inner and outer interfaces that happens to conserve the mass as the cross-sectional area between the two interfaces decreases with the outer drop contraction. Bigger nozzle causes the drop to grow bigger before it pinches,^28^ so when the inner nozzle increases, the overall drops weight increases allowing the outer drop to stretch downward under its weight leading to more contraction of the outer drop neck on the inner drop filament (see Figure 9a–c). This causes the focusing effect, which increases the inner drop thinning rate. The contraction increases also the stretching of the inner drop filament before the pinching, leading to longer filament length.

**Figure 9:**
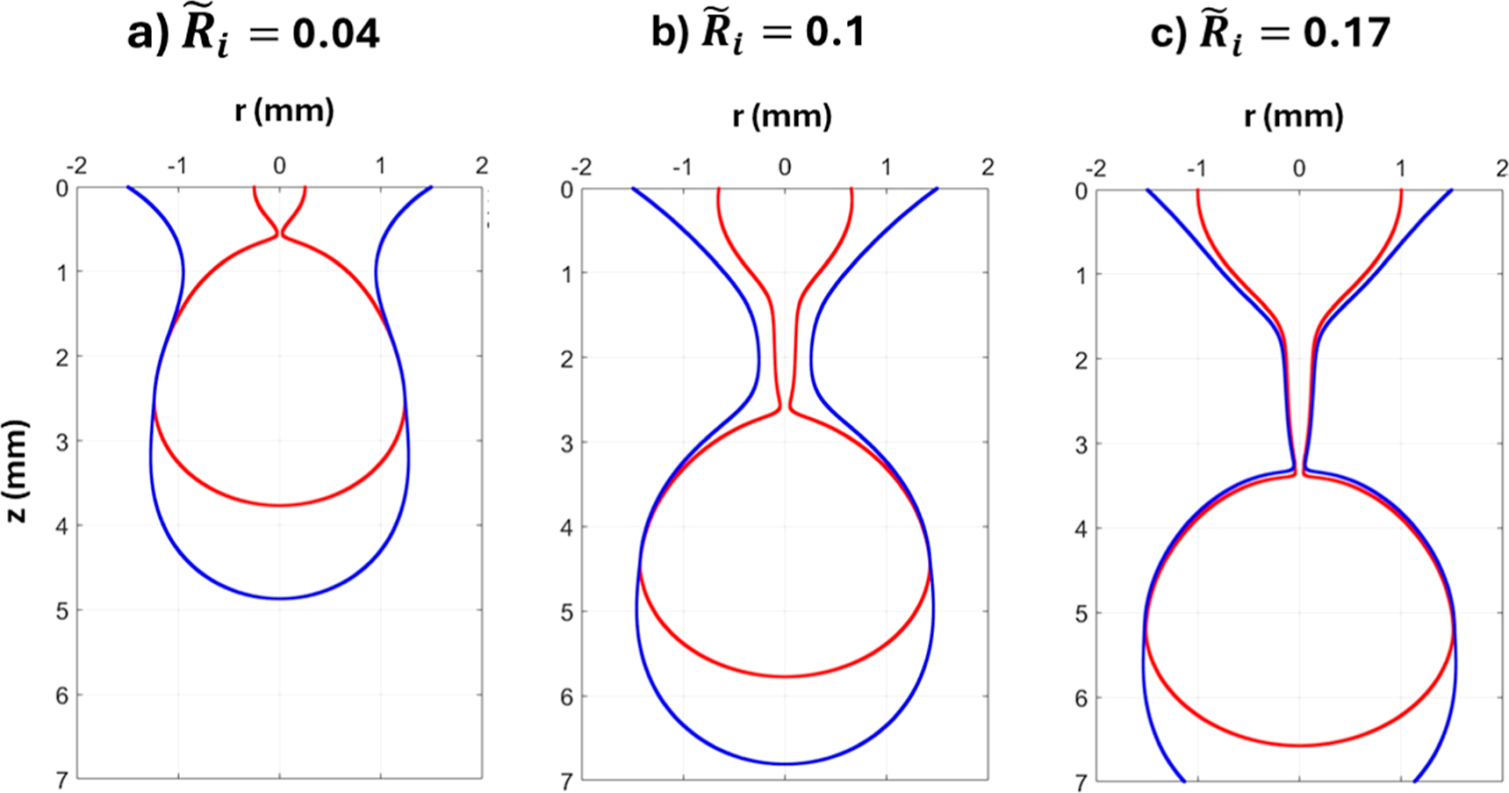
Coaxial drop pinching configurations at ${\mathrm{R̃}}_{o}=$ 0.25 for ${\mathrm{R̃}}_{i}$ of (a) 0.04, (b) 0.1, and (c) 0.17. The snapshots were taken at the moment of *A*_peak_.

The pressure and velocity contours shown in Figure 10 reveal how the focusing effect, produced by the outer interface when its neck is contracting, accelerates the rate of thinning. As the outer interface approaches the inner interface, the pressure in the inner neck region increases. This leads to a higher-pressure difference in the axial direction that accelerates the flow out of the neck, causing a higher thinning rate. This is evident from the velocity contour, which shows a higher axial velocity when there is more outer drop neck contraction. For ${\mathrm{R̃}}_{i}$ = 0.1, the contraction causes a maximum pressure at the inner neck of 414 Pa, while for ${\mathrm{R̃}}_{i}$ = 0.17, we see more contraction leading to a higher maximum pressure of 868 Pa.

**Figure 10:**
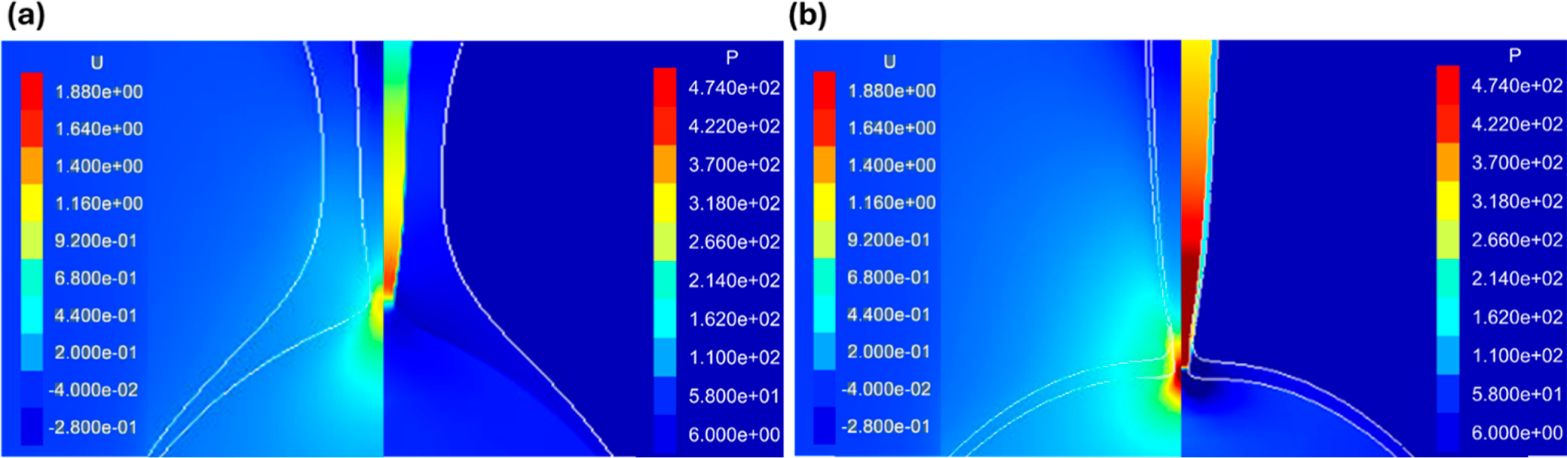
Side-by-side pressure and axial velocity distribution at the inertial prefactor peak time for (a) ${\mathrm{R̃}}_{i}$ = 0.1 and ${\mathrm{R̃}}_{o}$ = 0.25 and (b) ${\mathrm{R̃}}_{i}$ = 0.17 and ${\mathrm{R̃}}_{o}$ = 0.25.

Figure 11 reveals that for nozzle ratio $\mathrm{R̃}={\mathrm{R̃}}_{i}/{\mathrm{R̃}}_{o}$ between 0.37 and 0.67, the outer neck contraction rate becomes faster than the thinning of the inner neck. Once the outer neck reaches the inner filament, a squeezing happens at a contact point, leading to the formation of a bulb. The contours in Figure 11 show how the contraction of the outer neck causes a localized high pressure at a certain location above the minimum inner neck. This leads to a flow out of this contact location, creating a new inner neck. The bulb is then formed between this contact point and the original inner neck. Figure 11 indicates that this localized pressure increases as *R̃* increases from 0.37 to 0.67. Moreover, the distance between the contact point and the original minimum inner neck decreases as *R̃* increases, leading to the formation of smaller bulbs. After the pinching of the original inner neck, the other formed inner neck is also pinched, leading to the formation of a satellite droplet trapped inside the outer drop.

**Figure 11:**
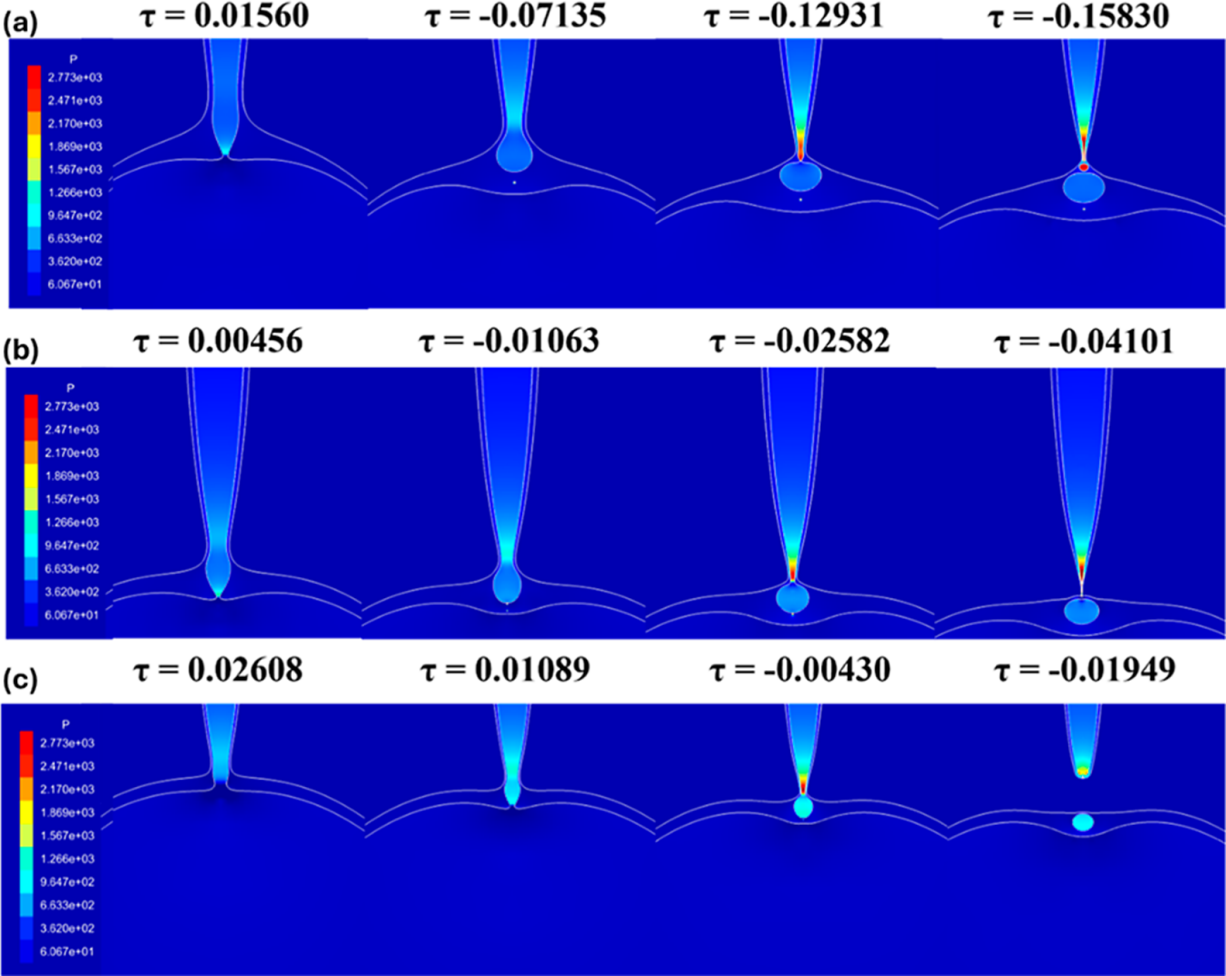
Pressure contours during outer drop contraction for (a) *R̃* = 0.37, (b) *R̃* = 0.57, and (c) *R̃* = 0.67.

Figure 8 illustrates that, for ${\overset{\tilde{}}{R}}_{i}>\;0.17$ , the thinning rate increases very rapidly before it reaches a maximum *A*_peak_ of 0.8. In this region, the contraction of the outer neck happens very close to the inner minimum neck (see Figure 12b), which accelerates the thinning in comparison with lower ${\mathrm{R̃}}_{i}$ (Figure 12a). Figure 8 also shows that *A*_peak_ becomes dependent on ${\mathrm{R̃}}_{o}$ when ${\mathrm{R̃}}_{i}>0.17$ . The thinning rate increases as ${\mathrm{R̃}}_{o}$ decreases, mainly because the outer interface becomes closer to the inner interface during the inner pinching (see Figure 12c,d). For *R̃* > 0.67, *A*_peak_ ranges between 0.5 and 0.8, which for higher *R̃* (>0.7) can surpass *A*_peak_ values found in literature for single-drop pinching, such as pinching in another liquid 0.66^23^ and pinching in air 0.6,^22^ including the theorized value of 0.7.^22^ This reflects the significance of the coaxial nozzle configuration to enhance and increase the thinning rate. Once the maximum *A*_peak_ of 0.8 is reached, at higher *R̃*, a resemblance between the interfaces (see Figure 12e,f) results in an identical thinning rate regardless of any higher *R̃*.

**Figure 12:**
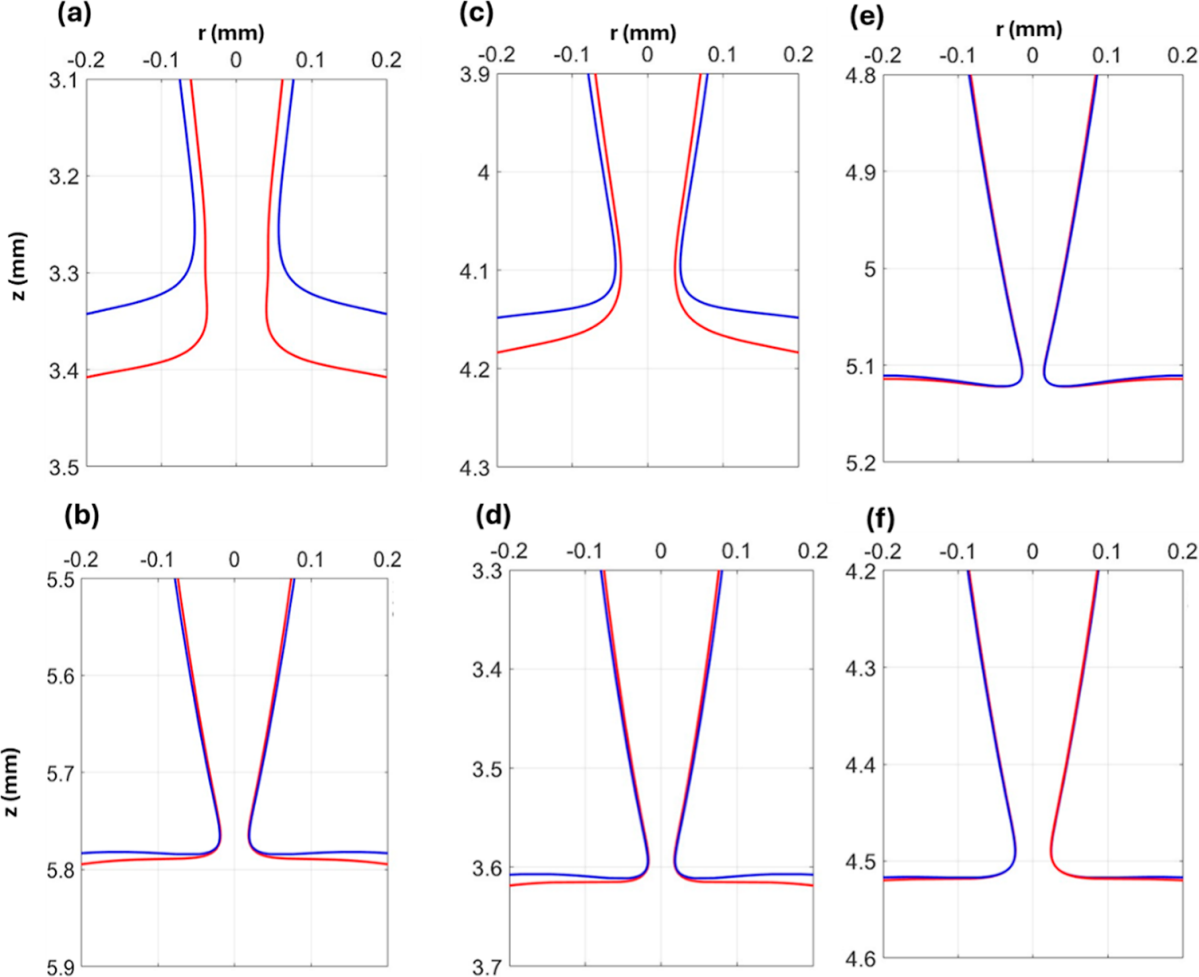
Zooming of the coaxial drop pinching configurations for (a) *R̃* = 0.67, (b) *R̃* = 0.75, (c) ${\mathrm{R̃}}_{i}=$ 1.2 and ${\mathrm{R̃}}_{o}=$ 1.75, (d) ${\mathrm{R̃}}_{i}=$ 1.2 and ${\mathrm{R̃}}_{o}=$ 1.5, and (e) *R̃* = 0.85 and (f) *R̃* = 0.91, at the moment of *A*_peak_.

Another aspect to explore is the temporal thinning nonlinear behavior. Figure 13 shows that as the nozzle ratio increases, the temporal inertial prefactor and hence the thinning rate distribution become more complex. For *R̃* < 0.4, the thinning rate increases to a peak point before it declines as the time approaches the pinching moment. This is similar to the inertial prefactor distribution in the pinching of a single drop case. However, for *R̃* > 0.4, a second peak of the thinning rate starts to appear (Figure 13a). This second peak reflects the focusing effect from the outer drop contraction when the outer drop neck starts to approach the inner drop filament, which leads to an increase in the inner drop thinning rate again. For *R̃* > 0.67, the increase in the second peak becomes significant (see Figure 13b) as the contraction of the external interface happens close to the minimum inner drop neck, as shown in Figure 12c,d.

**Figure 13:**
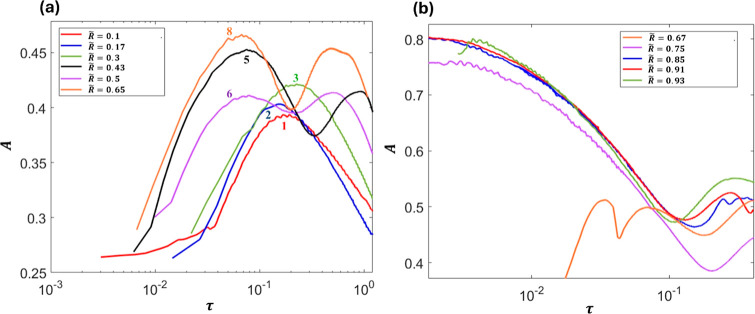
Temporal distribution of *A* for the inner drop when *R̃* is between (a) 0.1 and 0.65 and (b) 0.67 and 0.93.

In the coaxial configuration, we found that the thinning rate is affected not only by the nozzle radii but also by the viscosity of the outer liquid. Figure 14 represents the peak inertial prefactor as a function of the Ohnesorge number *Oh*_*o*_ of the outer liquid for different nozzle ratios, where ${Oh}_{o}=\mu /\sqrt{\left(\rho_{o}\sigma L_{c}\right)}$ . Here, we used the capillary length as the characteristic length to consider viscous effects independent of the external geometry. The results in Figure 14 show that the thinning rate of the inner drop decreases by increasing the outer liquid viscosity, in agreement with a previous study for single drop pinching in other liquids, as viscous force resists the flow, reducing the thinning rate.^23^ This viscous effect becomes less important at higher nozzle ratios, as the focusing effect of the outer interface on the inner neck becomes more dominant on the pinching process. This leads to a fast thinning with *A*_peak_ around 0.8. Figure 14b,c reveals a linkage between the inhibiting effect of the viscosity and the proximity between the inner and outer interfaces of the coaxial drops. As the viscosity increases, the interfaces are more distanced. This means less contraction on the inner neck and hence a lower thinning rate, in agreement with the pattern found in Figure 14a.

**Figure 14:**
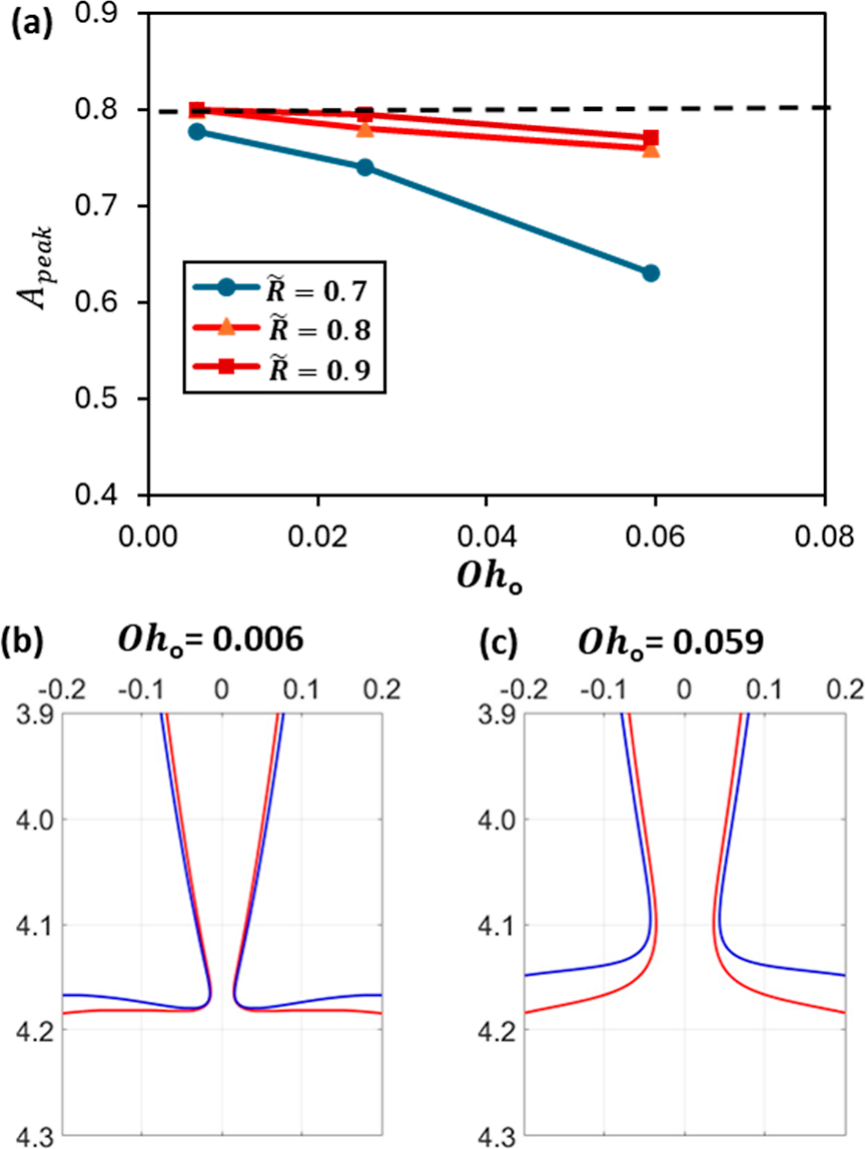
(a) Maximum inertial prefactor *A*_peak_ represented for liquids with different viscosities and nozzle radii and the zooming of the coaxial drop pinching configurations for (b) *Oh*_*o*_ = 0.006 and (c) *Oh*_*o*_ = 0.059.

From the previous discussions, we can conclude that the inner drop thinning rate is mainly affected by the proximity between the inner and the outer drop’s neck. Therefore, to reflect this, we define a characteristic parameter ${\Delta h}_{\min}=h_{\min outer}-h_{\min inner}$ (see Figure 15a), which is the difference between the inner and outer minimum neck radii. We can then use this parameter to explain the complexity of the temporal thinning behavior for the different cases. For *R̃*< 0.4, Δ*h*_min_ increases as the time to the pinching progresses (see Figure 15b). This means that the inner drop neck moves away from the outer drop neck, and there is no outer contraction effect on the inner thinning rate. As a result, we get the same single-peak behavior as if the inner drop is thinning in a single-nozzle configuration. However, we need to emphasize that *A*_peak_ is still higher than the single case because the outer drop weight pulls the inner drop downward. For *R̃* > 0.4, Δ*h*_min_ decreases with time as the outer drop thinning rate becomes faster (see Figure 15c). When Δ*h*_min_ becomes small enough, a focusing effect led by the outer drop neck contraction starts to appear. This causes the inner thinning rate to increase again, creating a second inertial prefactor peak. Because the inner thinning rate is now faster, Δ*h*_min_ starts to increase, as shown in Figure 15c. In cases where a bulb is formed in the inner drop filament (Figure 11), multiple inertial prefactor peaks might appear, as shown in Figure 15d. In this case, Δ*h*_min_ declines with a high rate, causing the appearance of a second peak. During this second peak, the inner minimum neck thinning becomes faster. However, $h_{\min\text{\_}outer}$ still declines causing a squeezing of the inner filament at the contact point. As a result, Δ*h*_min_ keeps decreasing, but at a slower rate. Once Δ*h*_min_ is small enough, a second focusing effect causes another inertial prefactor peak to appear. This is then followed by an increase in Δ*h*_min_ because the inner drop thinning becomes much faster. For *R̃* > 0.77, when the outer drop neck thins next to the inner drop neck, Δ*h*_min_ keeps declining, causing a second peak of the inertial prefactor with a significant increase (see Figure 15e).

**Figure 15:**
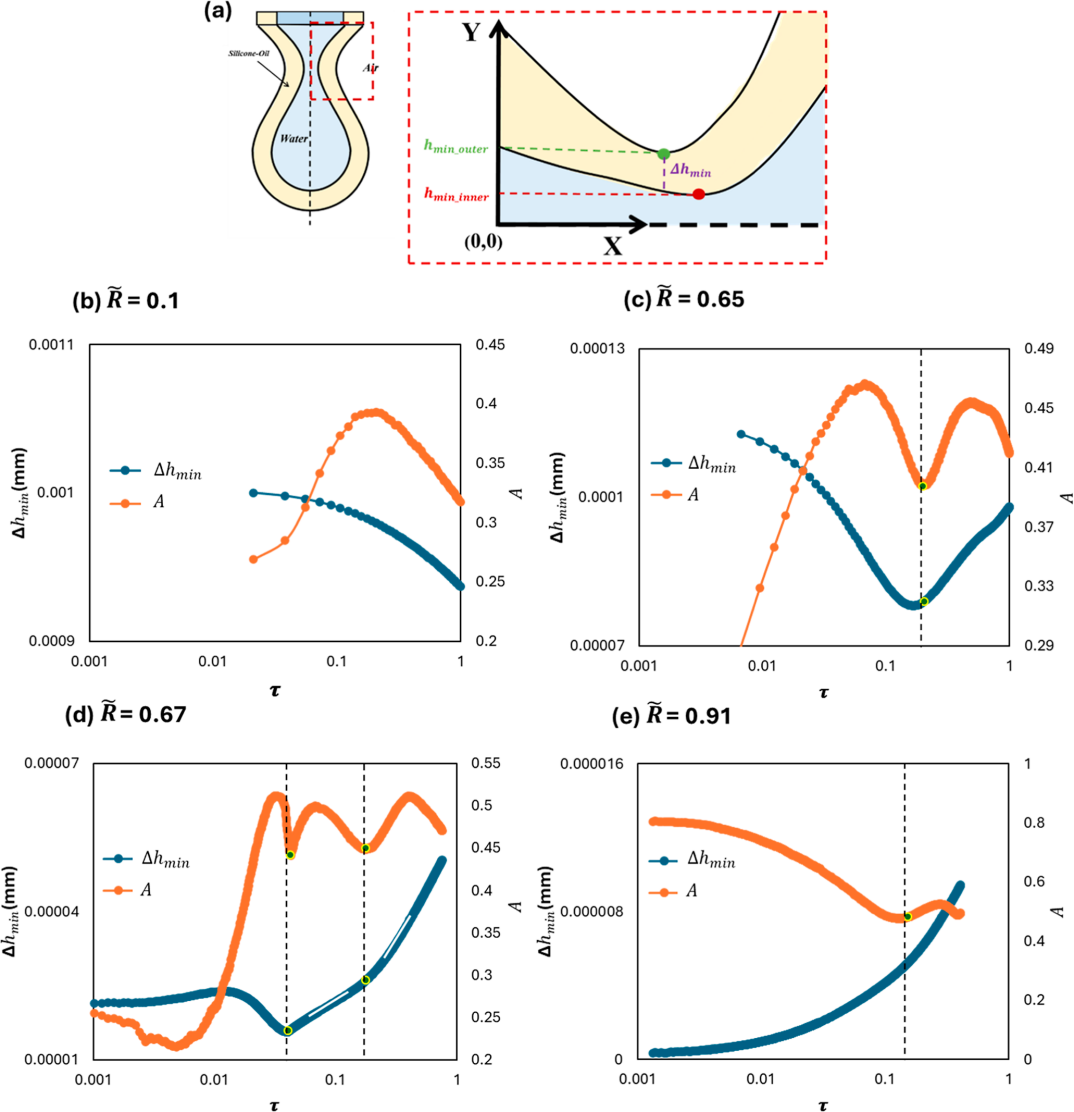
(a) Schematic showing the temporal inertial prefactor of the inner drop and its corresponding Δ*h*_min_ for *R̃* of (b) 0.1, (c) 0.65, (d) 0.67, and (e) 0.91.

Finally, we represent *A*_peak_ against its corresponding ${\Delta \mathrm{h̃}}_{\min}={\Delta h}_{\min}/L_{c}$ for all our data, which consider the effect of nozzle size and outer liquid viscosity. Figure 16 shows a nice collapse of all data in a monotonic relation. In general, the thinning rate represented in *A*_peak_ increases as ${\Delta \mathrm{h̃}}_{\min}$ decreases. This increase of *A*_peak_ is slow for ${\Delta \mathrm{h̃}}_{\min}$ > 0.01 and rapid for 0.0002 < ${\Delta \mathrm{h̃}}_{\min}$ < 0.01 because the outer drop’s neck approaches the inner drop’s filament close enough to squeeze it and rapidly increase the rate of thinning. Lastly, when Δ*h*_min_→ 0 $({\Delta \mathrm{h̃}}_{\min}$ < 0.0002), the thinning rate becomes independent of Δ*h*_min_, as shown in Figure 16, subgraph. The highlighted area in Figure 16, where 0.003 < ${\Delta \mathrm{h̃}}_{\min}$ < 0.01, indicates the region where satellite droplet formation occurs for a coaxial configuration. In this region, the outer drop neck squeezes the inner drop filament above the inner neck, causing the formation of a bulb that eventually pinches from both ends into a satellite droplet inside the outer drop. For very small ${\Delta \mathrm{h̃}}_{\min}$ below 0.003, the contraction of the outer drop neck happens very close to the inner drop neck. This does not allow for an inner drop double pinching, inhibiting the formation of a satellite droplet.

**Figure 16:**
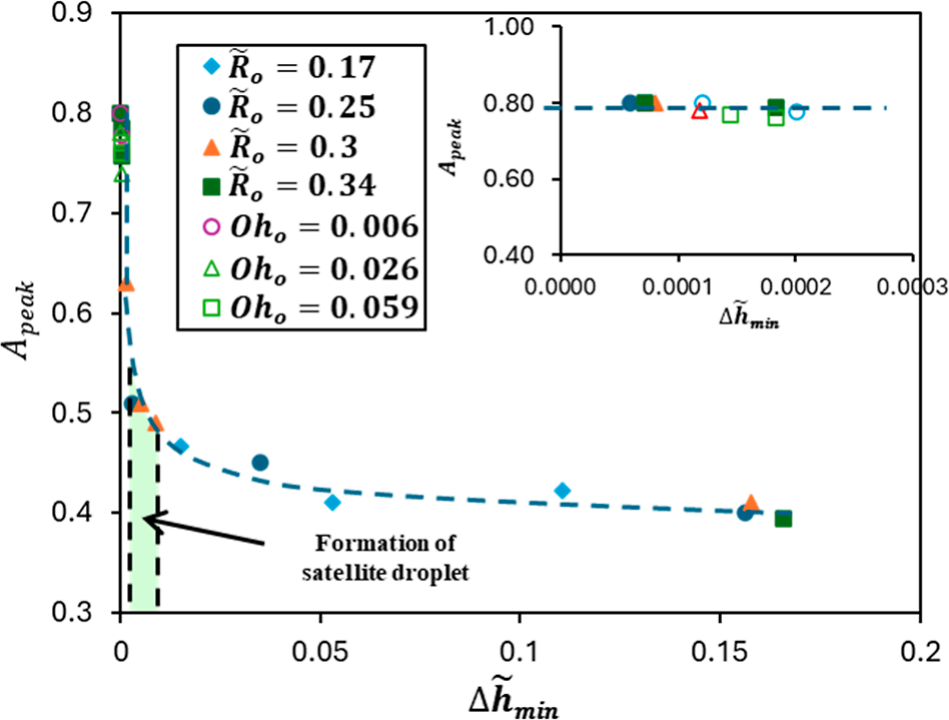
Variation of *A*_peak_ as a function of the corresponding ${\Delta \mathrm{h̃}}_{\min}$ for the inner drop. The section in green highlights the values of ${\Delta \mathrm{h̃}}_{\min}$ where encapsulated satellite droplets are produced within the outer drop after the pinching.

## Conclusions

In conclusion, our results demonstrate that the thinning rate of the inner drop in a coaxial dripping structure is faster than that of a single drop dripping into a liquid pool. Given that we consider in this study an inner drop with low viscosity, our results are more focused on the pinching in the inertial regime. As the outer drop begins to contract, the thinning rate of the inner drop increases gradually with the inner nozzle ${\mathrm{R̃}}_{i}$ , due to a focusing effect that increases the pressure at the neck of the inner drop, thereby accelerating fluid outflow. For *R̃* > 0.67, the necking of both the inner and outer drops occurs simultaneously, and the difference between inner and outer minimum neck radii Δ*h*_min_ continues to decrease. This results in a rapid increase in the thinning rate with increasing *R̃*. Also, for *R̃* > 0.67, the thinning rate decreases with the outer nozzle size before it reaches a constant *A*_peak_ of 0.8. In the region of constant *A*_peak_, the interfaces of the inner and outer drops remain matched with their minimum necks located at the same axial position from the nozzle. Consequently, the thinning rate becomes independent of *R̃*. Outer liquid drop viscosity is found to reduce the thinning rate at low *R̃*. As *R̃* increases, the viscosity becomes less effective, and the focusing effect from the outer drop contraction dominates, causing a thinning rate with *A*_peak_ ∼ 0.8. The temporal thinning behavior is found to be governed by Δ*h*_min_. For *R̃*<0.4, Δ*h*_min_ increases over time, producing a single peak in the temporal distribution of the thinning rate *A*. In contrast, for *R̃* > 0.4, Δ*h*_min_ decreases with time, leading to a squeezing of the inner drop filament and the emergence of multiple peaks in the temporal profile of *A*. Finally, the peak thinning rate of the inner drop, denoted as *A*_peak_, is expressed as a function of ${\Delta \mathrm{h̃}}_{\min}$ . For $0.003<{\Delta \mathrm{h̃}}_{\min}<0.01$ , the outer drop compresses the inner filament above its minimum neck, resulting in the formation of a satellite droplet encapsulated within the outer drop. This satellite regime also corresponds to a nozzle ratio *R̃* between 0.37 and 0.67. These findings provide valuable insights into the dynamics of coaxial liquid pinching and offer strategies for better controlling the process, including the prevention of satellite droplet formation during capsule generation. This has significant implications for applications in drug delivery, cosmetics, agriculture, and biomedical engineering.
